# SARS-CoV-2 Seropositivity among Dental Staff and the Role of Aspirating Systems

**DOI:** 10.1177/2380084421993099

**Published:** 2021-04

**Authors:** M. Sarapultseva, D. Hu, A. Sarapultsev

**Affiliations:** 1 Department of Pediatric Dentistry, Medical Firm Vital EVV, Ekaterinburg, Russia; 2 Institute of Immunology and Physiology (IIP) of the Ural Division of Russian Academy of Sciences, Ekaterinburg, Russia; 3 Department of Integrated Traditional Chinese and Western Medicine, Union Hospital, Tongji Medical College, Huazhong University of Science and Technology, Wuhan, China; 4 School of Medical Biology, South Ural State University, Chelyabinsk, Russia

**Keywords:** dental public health, dental health survey, infection control, infectious disease, severe acute respiratory syndrome coronavirus 2, virology

## Abstract

**Introduction:**

Health care workers (HCWs) are at a high risk of infection owing to occupational exposure to patients and virus-contaminated surfaces.

**Objectives:**

The study was aimed to reveal and compare the seroprevalence of severe acute respiratory syndrome coronavirus 2 (SARS-CoV-2) infection among patient-facing HCWs across 3 dental clinics equipped with different types of aspirating systems.

**Methods:**

This retrospective cohort study included 157 HCWs (43.58 ± 1.66 y) from 3 dental clinics in Ekaterinburg (Russian Federation) who reported to work during the coronavirus disease pandemic. All HCWs underwent serological testing once a week to detect immunoglobulin G and M antibodies against the SARS-CoV-2. The V6000 aspirating system with a vacuum controller (dry or semidry mode) and high-efficiency particulate air (HEPA) filters was used at clinics A and B, and the aspirated aerosol and air were evacuated and dissipated into the atmosphere. The VS900 aspirating vacuum pump without HEPA filters was used at clinic C. The aspirated aerosol and air were evacuated and dissipated into the operatories. All dental clinics followed the same recommendations for dental patient management and types of personal protective equipment used.

**Results:**

The estimated prevalence of SARS-CoV-2 infection was 11.5% (19 HCWs) over a 5-mo follow-up (May to August 2020). The prevalence of infection was unaffected by sex or the role of the member in the dental team (dentist/dental assistant). The prevalence of SARS-CoV-2 infection (+) was significantly higher at clinic C (equipped with an aspirating vacuum pump without HEPA filters) than at other clinics.

**Conclusion:**

The type of aspirating system used and the presence of HEPA filters could affect the prevalence of SARS-CoV-2 infection across dental clinics. Therefore, we recommend the use of aspirating systems installed with HEPA filters, which evacuate and dissipate aerosols into specialized areas.

**Knowledge Transfer Statement:**

This report confirms that dentists, being patient-facing HCWs, are at a high risk of acquiring the SARS-CoV-2 infection and identifies gaps in the protection of patients and staff in dental settings.

## Introduction

Health care workers (HCWs) are at a high risk of infection owing to occupational exposure to patients and virus-contaminated surfaces (Celebi et al. 2020; Meng et al. 2020; Reusken et al. 2020). The risk of exposure to coronavirus disease (COVID-19) is higher in HCWs in patient-facing roles than in their colleagues in non–patient-facing roles (Gómez-Ochoa et al. 2021; Hughes et al. 2020; Misra-Hebert et al. 2020), although the seropositivity rate is approximately 2-fold higher (13.2%) even in HCWs in non–patient-facing roles than in the general population (Razvi et al. 2020). From a subset job setting for HCWs with COVID-19, most medical personnel who showed positive results on COVID-19 testing were known to work in hospital/nonemergency wards (Gómez-Ochoa et al. 2021). The risk of severe acute respiratory syndrome coronavirus 2 (SARS-CoV-2) transmission is likely to be higher in dentists than in other HCWs (US Department of Labor 2020), which is attributable to the fact that dental health care delivery requires close physical contact between patients and specialists, and dental procedures generate aerosols, which may pose potential risks to operators and patients (Ge et al. 2020; Kutter et al. 2018). Reportedly, 15-min treatment without protective measures can result in inhalation of approximately 0.014 to 0.12 µL of saliva via the aerosol (Bennett et al. 2000), which can travel beyond 6 feet and spread virus particles, as shown in cases of SARS-CoV-2 infection (Kutter et al. 2018). Although no reports have described COVID-19 cases transmitted in a dental setting, SARS-CoV-2 may remain suspended in the environment for more than an hour. Notably, aerosols can contaminate not only the specific area of care but also the entire hospital environment (de Almeida Barros Mourão et al. 2020; Ge et al. 2020). Moreover, the estimated median half-life of SARS-CoV-2 is approximately 5.6 h on stainless steel and 6.8 h on plastic surfaces (van Doremalen et al. 2020), which further increases the risk of infection.

Working in high-risk departments, suboptimal handwashing practices before and after patient contact, longer working hours, and inappropriate use of personal protective equipment (PPE) were risk factors associated with SARS-CoV-2 transmission among HCWs (Ran et al. 2020). Based on these observations, measures have been suggested for dental practitioners to contain the spread of COVID-19 (Centers for Disease Control and Prevention 2020b; Ge et al. 2020; Goswami et al. 2020; Izzetti et al. 2020). Most guidelines describe the following measures that can be adopted by dentists and dental hygienists to reduce aerosol formation: scaling and root planing procedures performed with handheld instruments rather than using ultrasonic scaling, use of minimally invasive dental procedures such as silver diamine fluoride application for caries treatment or the Hall technique for badly broken primary teeth among pediatric patients, and the use of high-velocity suction and rubber dental dams whenever possible (Centers for Disease Control and Prevention 2020b; Izzetti et al. 2020). No published data have described the role of the type of aspirating system as a risk factor for SARS-CoV-2 infection among dental specialists. In this pilot study, we investigated and compared the prevalence of SARS-CoV-2 infection among patient-facing HCWs across 3 dental clinics equipped with different types of aspirating systems.

## Materials and Methods

This retrospective cohort study was performed during the COVID-19 pandemic between May and August 2020 across 3 dental clinics (2 private clinics and 1 government center) in Ekaterinburg (Russian Federation) serving for medical treatment during the COVID-19 outbreak. The study included dentists or dental assistants from the designated hospitals. The study was performed in accordance with the Declaration of Helsinki of 1975, revised in 2013. Ethical approval C-20-04-2020 (April 20, 2020) was obtained from the Institute of Immunology and Physiology of the Ural Division of Russian Academy of Science, Ekaterinburg, and informed consent was obtained from all HCWs recruited for the study. Strengthening the Reporting of Observational Studies in Epidemiology guidelines were followed to report this study.

### Study Population

The study included 157 patient-facing HCWs recruited from 3 dental clinics who reported to work during the COVID-19 pandemic. HCWs aged >65 y, those with chronic diseases or immunological disorders, and pregnant women were not permitted to work during the pandemic based on the National Recommendations (Appendix) and were excluded from the study. All HCWs underwent serological testing once a week to detect immunoglobulin (Ig) G and IgM antibodies against SARS-CoV-2 based on the National Recommendations (Appendix). Antiviral antibodies were detected using the SARS-CoV-2-IgG-EIA-BEST and SARS-CoV-2-IgM-EIA-BEST enzyme immunoassay kits (VECTOR-BEST).

### Dental Patient Management

Dental treatment during the pandemic was limited to emergency and urgent cases (Russian Dental Association 2020). The National Regulations of the time required all dental specialists and patients to follow the social distancing protocols. Based on the Ministry of Health order (198n, dated March 19, 2020), 2-step patient management comprising a remote and face-to-face (patient admission) approach had to be employed. Remote management included obtaining information regarding patients’ COVID-19 history and patients’ status (an established diagnosis of COVID-19 or contact with a person with COVID-19 in the past 14 d, symptoms of acute respiratory diseases, pregnancy status, and age >65 y) via questions answered by patients (Appendix). Based on the responses obtained from patients, they were recommended to postpone their visit until the end of the quarantine period (14 d) or were scheduled for an appointment (those in whom emergency medical intervention was indicated). Appointments were scheduled to ensure that only a single patient would visit the clinic at the designated appointment. Patients who visited without a mask were provided a surgical mask, their hands were disinfected, and their body temperature was recorded using infrared thermometers. The interview was then repeated in written form.

### Personal Protective Equipment Usage and Hand Disinfection

According to the National Regulations, all the specialists were subjected to use PPE and perform hand hygiene. The reinforced type II PPE set consisted of a medical suit (similar to pajamas), a large surgical disposable gown and cap, a respirator (N95/FFP2), a face shield or safety glasses, disposable rubber gloves, socks, waterproof high shoe covers, and disposable towels. Hand disinfection was performed using alcohol-based (70%) solutions.

### Aspirating Systems and Office Area

The area of dental operatories was the same across all clinics included in this study (not less than 14 m^2^, based on sanitary regulations). Aspirating systems used by all clinics were tested at least once a month using a vacuum tester (based on the sanitary regulations). The standard suction power used was 310 to 315 L/min.

The aspirating central vacuum pump V6000 (DÜRR DENTAL AG; Bietigheim-Bissingen) and the aspirating vacuum pump VS900 were used for 3 workstations (DÜRR DENTAL AG) (Dürr Dental 2020) in this study. The V6000 aspirating system is a central aspiration machine with a vacuum controller (dry mode or semidry mode) and a bacterial filter. This system can be used by a maximum of 20 operators at a time across up to 30 workplaces. The aspirated aerosol and air were evacuated into a special area in the basement of the clinic and later dissipated in the atmosphere. The VS900 is an aspirating vacuum pump (without a HEPA filter) that can be used at 3 workstations. The aspirated aerosol and air were evacuated into the dental operatories and later dissipated into the atmosphere of the operatory.

### Patient and Public Involvement

Neither patients nor the public were involved in the conception or implementation of the study.

### Data Analysis

Data management and analysis were performed using the SPSS software, version 15.0 (SPSS, Inc.). Graphs and charts were prepared using the Microsoft Excel statistical software, version 14.0. Pearson’s χ^2^ test (Yates’s correction) was used for analysis of categorical variables.

## Results

This study included 157 HCWs employed at 3 clinics. Of these, 119 (75.8%) HCWs were women, and nearly 50% (49.7%) of all HCWs were dental specialists (dentists). The mean participant age was 43.58 ± 1.66 years. The V6000 aspirating system was used in clinics A and B, with the drying mode–dry mode used in clinic A and the semidry mode in clinic B. The VS900 aspirating vacuum pump was used in clinic C. The reinforced type II PPE set (without the second pair of gloves) was used at all clinics (Table).

**Table.** HCWs’ Distribution and Working Conditions.

Clinic	Gender	Count	Mean Age ± CI	Dentist	Dental Assistant	Aspiration System	PPE Set	HEPA Filters
A	Female	43	42.74 ± 3.19	28	28	V6000 (dry mode)	Reinforced type II	H14
	Male	13	47.46 ± 9.22					
B	Female	46	43.15 ± 2.77	30	30	V6000 (semidry mode)	Reinforced type II	H14
	Male	14	46.36 ± 7.3					
C	Female	30	42.43 ± 3.2	20	21	VS900	Reinforced type II	None
	Male	11	43.73 ± 8.03					
Total		157	43.58 ± 1.66	78	79			

H14 HEPA (EN 1822-1:2019-10/ISO 45H/ISO 29463-1:2017).

The estimated prevalence of SARS-CoV-2 infection was 11.5% (19 HCWs) over the 5-mo follow-up (May to August 2020). Analysis performed using Pearson’s χ^2^ test with Yates’s correction showed that the prevalence of SARS-CoV-2 infection was not associated with sex (χ^2^ = 0.394, *df* = 1, *P* = 0.53, power = 0.09) or the role of the member in the dental team (dentist/dental assistant; χ^2^ = 0.211, *df* = 1, *P* = 0.646, power = 0.07) (Figure).

**Figure.**
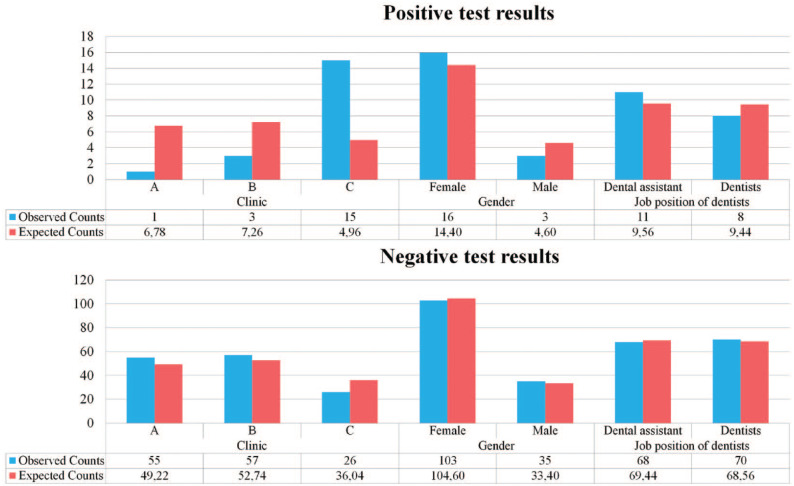
Test results for severe acute respiratory syndrome coronavirus 2 infection.

The most significant differences in the prevalence of SARS-CoV-2 infection were recorded between clinics A and C (χ^2^ = 31.552, *df* = 2, *P* < 0.001, power = 0.99) (Figure). The number of SARS-CoV-2 infections (+) was significantly higher in clinic C than in clinics A and B and significantly lower in clinic A.

## Discussion

The estimated seroprevalence of SARS-CoV-2 infection among the HCWs investigated in this study was 11.5% (19 HCWs) over the 5-mo follow-up (May to August 2020). No data are available regarding SARS-CoV-2 seroprevalence among dental specialists, and data on SARS-CoV-2 infection among dental professionals vary significantly across countries (Estrich et al. 2020; Meng et al. 2020). The seroprevalence of SARS-CoV-2 infection among dentists in the United States was 0.9% to 1.7% (Estrich et al. 2020; Hughes 2020; IFHD 2020). A significantly higher prevalence (approximately 25%) was reported in Mexico (Antonio-Villa et al. 2020). These variations can be attributed to but are not limited to the time point of testing, variations in HCW job types, PPE usage guidance, and the differences between various antibody assays used in studies (Razvi et al. 2020). The size of dental clinics and the number of staff can also affect the prevalence of SARS-CoV-2 infection. Most general dental clinics are relatively small in the United States, Europe, and China, with the mean number of dentists and dental hygienists per practice being 1.5 and 0.8, respectively, in the United States (Le and Lo Sasso 2020) and 3.4 dentists/hygienists in China (Meng et al. 2020). In contrast, most dental clinics in Russia and Ukraine are large dental centers and include various specialists (orthodontists, orthopedists, surgeons, pediatric dentists, periodontists, and general practitioners, among other specialties) within 1 medical organization (Analytical Center VADEMEDICUM 2016; Slipchenko 2017). Therefore, it is more appropriate to compare the prevalence of SARS-CoV-2 observed in the present study with that observed among HCWs working in large-sized hospitals. Our results are similar to those reported by a meta-analysis performed by Gomez-Ochoa et al. in 2021; however, the prevalence of SARS-CoV-2 infection in our study was higher than that reported by other studies, which observed that 6% to 9% of all HCWs showed COVID-19 seropositivity (Consonni et al. 2020; Self et al. 2020). The seroprevalence rate of SARS-CoV-2 infection among dental health care workers in this study (11.5%) is higher than in the general population elsewhere in the Russian Federation (7.4%–9.3%) (Barchuk et al. 2020). This may indicate that dental health care workers are at increased risk of SARS-CoV-2 infection or may be a result of geographic and temporal differences. Moreover, because of regulatory rules, individuals aged >65 y were not permitted to work during a pandemic and were excluded from this study. The risk of COVID-19 infection increases with age; therefore, the estimated prevalence of SARS-CoV-2 infection could have been underestimated in this study.

In contrast to the findings reported from other countries, the prevalence of SARS-CoV-2 infection observed in this study was not associated with sex (Consonni et al. 2020; Razvi et al. 2020) or position/role in the dental team (Gómez-Ochoa et al. 2021). The lack of a significant association with sex is consistent with previous population-based studies (Barchuk et al. 2020; Popova et al. 2020) and could also be attributed to the insufficient power of statistical tests used. However, both dentists and dental assistants are in close contact with patients during treatment, which may explain the lack of differences in the risk of infection between these HCW categories.

The results of the present study revealed significant differences across clinics with regard to the ratio of positive results on SARS-CoV-2 antibody testing. The number of COVID-19+ patients was significantly higher in clinic C than in clinics A and B and significantly lower in clinic A (Figure). SARS-CoV-2 antibody test results were positive in 15 HCWs in clinic C (36.59% of tests showed positive results), whereas only 1 and 3 (1.79% and 5.0% of test results) were positive in clinics A and B, respectively. The area of the dental operatories, dental patient management, treatment protocols, mean age of HCWs, working schedules, and types of PPE used did not differ between clinics. Although we could not exclude differences in actual PPE use, nonclinical staff interactions, or dental patient management adopted in real-world practice, the types of aspirating systems installed significantly differed between clinics, which could affect the risk of SARS-CoV-2 infection.

The most pronounced difference between the aspirating systems used is that the VS900 system discharges air into the dental operatory, closely resembling natural dispersion, whereas the V6000 system discharges air into the external environment, which could account for the significantly higher positive antibody test results observed in clinic C (equipped with the VS900 system) than in clinics A and B. High-volume aspirators that minimize droplets and aerosol during high-speed turbine operations are recommended during the COVID-19 pandemic (Luzzi et al. 2021); however, no comparative studies have investigated the effects of the type of aspirating system on the risk of SARS-CoV-2 infection among HCWs. Our results indicate that these data are important because the type of aspirating system has been shown to significantly affect the incidence of SARS-CoV-2 infection among dental specialists; therefore, studies that provide a deeper understanding of this topic are warranted.

The use of HEPA filters could have significantly affected the differences in SARS-CoV-2 antibody test results observed between the clinics. H14 HEPA filters were used in aspirating systems in clinics A and B in contrast to aspirating systems without HEPA filters used in clinic C. HEPA filters can remove 99.97% of particles measuring 0.3 µm in diameter (Schentag et al. 2004; Christopherson et al. 2020). Although no study has reported an association, if any, between HEPA filtration and SARS-CoV-2 infection, their efficacy in capturing particulate matter of similar size and role in disease containment is well documented (Schentag et al. 2004). Therefore, installation of HEPA purifiers should be considered an adjunctive infection control measure for SARS-CoV-2 (Centers for Disease Control and Prevention 2020s; Christopherson et al. 2020; Schentag et al. 2004).

Finally, an important factor that could have affected the differences in results observed between clinics A and B is the type of suction unit installed in the aspirating system. The aspirating system functioned in the dry mode in clinic A as opposed to the semidry mode used in clinic B. In dry suction systems, the separation of aspirated fluids from the air occurs at every treatment unit, whereas in semidry suction systems, such separation occurs via a central separation unit, which is connected to multiple treatment units (Dürr Dental 2020).

Large quantities of the SARS RNA detected in patients’ saliva during the coronavirus outbreak in 2003 suggested the possibility of coronavirus transmission through oral droplets (Wang et al. 2004). Later, angiotensin-converting enzyme 2 expression was observed in the oral mucosa and epithelial cells of the tongue (Xu et al. 2020), which explains the mechanism of entry of SARS-CoV-2 through the oral cavity, confirming that the mouth is a potentially high-risk route of transmission (Xu et al. 2020). Therefore, based on the principle of universal precautions, special measures targeted toward minimizing aerosol transmission are warranted (Ge et al. 2020). Considering the significance of aerosol transmission and its implications in dentistry, the Centers for Disease Control and Prevention (CDC) and the CDC Division of Oral Health and dental specialists recommend the installation of HEPA filters in air filtration units for safety during and immediately following an aerosol-generating procedure (Centers for Disease Control and Prevention 2020b; Jamal et al. 2020; Silva et al. 2020). Although the use of high-volume saliva ejectors was recommended in addition to the universal precautions (Ather et al. 2020; New Zealand Dental Association 2020), no recommendations are available regarding the use of HEPA filters in aspirating systems.

## Conclusion

According to the results of the present pilot study, the overall seroprevalence rate among the patient-facing HCWs of 3 dental clinics was 11.5%. With that, significant differences in HCWs’ seroprevalence rate were observed among the dental clinics, subjected to the common regulatory requirements but equipped with different types of aspirating systems. Although we cannot exclude significant differences in actual real-world practice, the type of aspirating system may have influenced the risk of aerosol transmission of SARS-COV-2. Thus, special precautionary measures targeted toward aerosol transmission should be taken, and the use of aspirating systems with HEPA filters, which evacuate air at the special area and dissipate in the atmosphere, can be recommended.

Taking into account the limitations of the present study, further large-scale prospective cohort studies that include ethnically and geographically diverse cohorts are warranted to gain a better understanding of the prevalence of and risk factors associated with SARS-CoV-2 infection among dental specialists.

## Study Limitations

Due to the retrospective nature of the study and the scale of the COVID-19 pandemic, this study has several limitations: 1) All data were obtained from HCW cohorts admitted in only 3 dental clinics in Ekaterinburg, Russia. Therefore, the revealed effects may be different among HCWs in geographically diverse populations and are not generalizable. Moreover, based on the regulation rules, HCWs aged >65 y were not permitted to work during a pandemic, which could affect the prevalence of infection observed in this study. 2) Information regarding the prehospital status of HCWs (which could be associated with numerous clinical parameters and risk factors for SARS-CoV-2 infection) was unavailable. 3) Owing to community transmission and lack of contact tracing of infections, it was not possible to infer whether HCWs were infected at a dental clinic or from some other source. 4) Although uniform guidelines and regulations were recommended for all clinics included in this study, we cannot exclude significant differences in actual PPE use or dental patient management policies adopted in real-world practice. Dental professionals’ use of PPE and the actual daily procedures they followed were not closely monitored; therefore, the results of this study should be interpreted with caution.

## Author Contributions

M. Sarapultseva, A. Sarapultsev, contributed to conception, design, data acquisition, analysis, and interpretation, drafted and critically revised the manuscript; D. Hu, contributed to conception and data interpretation, drafted and critically revised the manuscript. All authors gave final approval and agree to be accountable for all aspects of the work.

## Supplemental Material

sj-pdf-1-jct-10.1177_2380084421993099 – Supplemental material for SARS-CoV-2 Seropositivity among Dental Staff and the Role of Aspirating Systems Click here for additional data file. Supplemental material, sj-pdf-1-jct-10.1177_2380084421993099 for SARS-CoV-2 Seropositivity among Dental Staff and the Role of Aspirating Systems by M. Sarapultseva, D. Hu and A. Sarapultsev in JDR Clinical & Translational Research
