# Supplementary material for: SARS-CoV-2 Seropositivity among Dental Staff and the Role of Aspirating Systems
Source: JDR Clin Trans Res. 2021 Feb 5;6(2):132–8. doi: 10.1177/2380084421993099 (PMC7868347; doi:10.1177/2380084421993099)
Supplement: sj-pdf-1-jct-10.1177_2380084421993099 – Supplemental material for SARS-CoV-2 Seropositivity among Dental Staff and the Role of Aspirating Systems [file sj-pdf-1-jct-10.1177_2380084421993099.pdf]

APPENDIX. REGULATORY DOCUMENTS.

- Decree of the Chief Sanitary Physician of Russian Federation No. 7 dated March 18, 2020 "On ensuring the isolation regime in order to prevent the spread of COVID- 2019 " [http://publication.pravo.gov.ru/Document/View/0001202003190001];
- Letter of Rospotrebnadzor dated 11.04.2020 N 02/6673-2020-32 "On the direction of recommendations on the use of PPE for various categories of citizens with the risk of infection with COVID-19" [https://base.garant.ru/73917166/]
- MR 3.1.0170-20.3.1. Prevention of infectious diseases. Epidemiology and prevention of COVID-19. Methodical recommendations" (approved by the Chief State Sanitary Physician of the Russian Federation on 03/30/2020, revised on 04/30/2020) [https://www.rospotrebnadzor.ru/region/korono_virus/files/spec/MR%203.1.0170-20.pdf]
- Order of Ministry of Health of Russian Federation from March 19, 2020 No. 198n" On the temporary procedure for organizing the work of medical organizations in order to implement measures to prevent and reduce the risks of the spread of a new coronavirus infection COVID-2019" [https://edu.rosminzdrav.ru/fileadmin/user_upload/specialists/COVID-19/Prikaz_Minzdrava_Rossii_ot_19.03.2020_N_198n__red._ot_27.03.2020g.__ver1.pdf]
- Prevention of the importation and spread of COVID-19 in medical organizations. Temporary guidelines. Version 2. Dated 05/14/2020. [http://nasci.ru/?id=10658&download=1].
- Recommendations from the Russian Federal Service for Surveillance on Consumer Rights Protection and Human Well-being to employers on compliance with the sanitary and epidemic regime, under which it is possible to carry out the economic activities No. MP-3.1/2.2.0170/3-20 dated April 06 [https://www.rospotrebnadzor.ru/files/news/%D0%9C%D0%A0%200170_3%20%D1%81%D1%80%D0%B5%D0%B4%D0%B8%20%D1%80%D0%B0%D0%B1%D0%BE%D1%82%D0%BD%D0%B8%D0%BA%D0%BE%D0%B2.PDF];
- Recommendations of the Chief State Sanitary Physician of Russian Federation on the prevention of a new coronavirus infection (COVID-19) No. 02/3853-2020-27 dated March 10, 2020 [https://www.rospotrebnadzor.ru/upload/iblock/cd1/10.03.2020_02_3853_2020_27_popova_a.yu._apparat_pravitelstva_respubliki_bashkortostan.pdf]
